# Subjective Sequelae in Cases of Brain Wounds and the Commotional Syndrome

**Published:** 1919

**Authors:** 


### Subjective Sequelae in Cases of Brain Wounds and the Commotional Syndrome. By A. Mairet and H. Pieron. *La Presse Médicale*, September 26, 1918.

The whole shock endured by the brain rather than the wound itself seems responsible for the subjective sequelae usually observed after brain wounds. Such was the author's conclusion in June, 1915, and this observation has been recently confirmed by MM. Pitres and Marchand.

The typical commotional syndrome exists as well in cases of shock with no wounds at all, as in cases of severe wounds of the head.

The principal features of this syndrome are : disturbances of sensibility, tendinous and muscular hyperexcitation, constant breaks in memory, and great difficulty in fixing the patient's attention on a given subject.

Such symptoms have been described lately by Prof. Grasset under the name of " atopical symptoms " in cases of brain wounds.

MM. Mairet and Piéron insist that all the principal features of shock, of " commotional syndrome ", are constantly met with among the subjective sequelae of the brain wounds, and also that they were the first to call medical attention to that subject.

